# Rationale for Involved Field Stereotactic Body Radiation Therapy-Enhanced Intermittent Androgen Deprivation Therapy in Hormone-Sensitive Nodal Oligo-Recurrent Prostate Cancer Following Prostate Stereotactic Body Radiation Therapy

**DOI:** 10.3389/fonc.2020.606260

**Published:** 2021-01-18

**Authors:** Michael Carrasquilla, Michael L. Creswell, Abigail N. Pepin, Edina Wang, Matthew Forsthoefel, Mary McGunigal, Elizabeth Bullock, Siyuan Lei, Brian T. Collins, Jonathan W. Lischalk, Giuseppe Esposito, Nima Aghdam, Deepak Kumar, Simeng Suy, Paul Leger, Ryan A. Hankins, Nancy A. Dawson, Sean P. Collins

**Affiliations:** ^1^Department of Radiation Medicine, Georgetown University Hospital, Washington, DC, United States; ^2^Georgetown University School of Medicine, Washington, DC, United States; ^3^George Washington University School of Medicine, Washington, DC, United States; ^4^Department of Nuclear Medicine, Georgetown University Hospital, Washington, DC, United States; ^5^Department of Radiation Oncology, Beth Israel Deaconess Medical Center, Boston, MA, United States; ^6^Biotechnology Research Institute, North Carolina Central University, Durham, NC, United States; ^7^Department of Oncology, Lombardi Comprehensive Cancer Center, Georgetown University Medical Center, Washington, DC, United States; ^8^Department of Urology, Georgetown University Hospital, Washington, DC, United States

**Keywords:** prostate cancer, involved field SBRT, intermittent ADT, Nodal Oligo-recurrence, hormone sensitive, prostate SBRT

## Abstract

Lymph node recurrent prostate cancer is a common clinical scenario that is likely to increase significantly with the widespread adoption of novel positron emission tomography (PET) agents. Despite increasing evidence that localized therapy is disease modifying, most men with lymph node recurrent prostate cancer receive only systemic therapy with androgen deprivation therapy (ADT). For men who receive localized therapy the intent is often to delay receipt of systemic therapy. Little evidence exists on the optimal combination of local and systemic therapy in this patient population. In this hypothesis generating review, we will outline the rationale and propose a framework for combining involved field SBRT with risk adapted intermittent ADT for hormone sensitive nodal recurrent prostate cancer. In patients with a limited number of nodal metastases, involved field stereotactic body radiation therapy (SBRT) may have a role in eliminating castrate-resistant clones and possibly prolonging the response to intermittent ADT. We hypothesize that in a small percentage of patients, such a treatment approach may lead to long term remission or cure.

## Androgen Deprivation Therapy For Lymph Node-Positive Prostate Cancer Following Radical Prostatectomy

The prognosis for men with lymph node-positive prostate cancer is superior to patients with bone metastases (1–4). Survival varies depending on the timing, location, and extent of disease (5). The optimal treatment strategy for these patients remains an area of active investigation. While most patients receive androgen deprivation therapy (ADT) as the standard of care, the ideal timing for the initiation of ADT (immediate versus delayed until time of symptoms) remains controversial. The major prospective randomized trials guiding this debate in post-prostatectomy patients provide conflicting evidence and were conducted prior to the advent of widespread PSA monitoring making it unclear if their findings still apply to today's patient population (6, 7). Results from a large national database, suggest that the prostate-cancer specific survival and overall survival are similar between immediate and delayed ADT in a patient population with PSA monitoring (8). Based on data such as this, the National Comprehensive Cancer Network (NCCN) and the International Society of Geriatric Oncology (SIOG) guidelines currently recommend immediate ADT but observation remains an option for the well informed asymptomatic patient who will be closely monitored (9, 10).

## Intermittent Androgen Deprivation Therapy

Side effects of ADT include hot flashes, decreased libido, fatigue, osteoporosis, weight gain, sarcopenia, and increased risk of cardiovascular disease. In general, side effects from ADT increase with the length of treatment duration (11, 12). Intermittent ADT (I-ADT) is a treatment option for men with biochemically recurrent prostate cancer with the aim of decreasing long term side effects from ADT (9, 13). Multiple large randomized trials comparing continuous ADT (C-ADT) to intermittent ADT in the metastatic setting have showed potential small improvements in overall survival with continuous ADT but improved quality of life (QOL) in the I-ADT arm (14–16). Likewise, multiple meta-analyses confirmed an improvement in quality of life for patients receiving intermittent ADT (17–19). Intermittent ADT requires close monitoring with regular PSA and total testosterone tests. Despite the increase in laboratory testing, patients on I-ADT require approximately one-third fewer LHRH injections compared to patients on continuous ADT. This leads to an overall cost savings of approximately 48% for patients receiving intermittent therapy (13, 18). With the advent of additional effective therapeutic agents in the metastatic setting, appropriate patient selection for intermittent ADT is critical (19). Patients with isolated lymph node metastases achieve longer remissions and are therefore most likely to benefit from this treatment strategy (13, 20). Currently, there are many unanswered questions regarding the logistics of administering intermittent ADT. These include the criteria for discontinuing/re-initiating ADT and which patients benefit most from transitioning from intermittent to continuous ADT. Past trials have used hormone responsiveness as a criterion for initiation and discontinuation of ADT without strong rationale for specific PSA cut-offs (21).

## Rationale For The Treatment of Nodal Oligo-Recurrent Disease

Prostate cancer has a tropism for lymph nodes, making it the second most common and with advanced PET imaging, frequently the only site of disease recurrence (22). Early in the natural history of the disease, nodal metastases are small with a median size of approximately 1 cm, which limits detection by standard imaging (< 2 cm) (23, 24). However, newer PET agents overcome many of these limitations. Imaging with ^68^Ga - prostate specific membrane antigen PET/CT (PSMA-PET) allows for the detection of small lymph node metastases at low PSA levels, shortening the time between biochemical and clinical recurrence (21). Using PSMA-PET imaging, greater than 50% of men with early PSA progression (≤ 0.5 ng/ml) have radiographic evidence of recurrence with >50% of those having abdominopelvic nodal involvement (25, 26). The radiographic detection rate of PSMA-PET continues to improve with increasing PSA levels (25–27).

With the advent of new sensitive and specific imaging the group of patients with oligo-recurrent nodal prostate cancer is likely to increase significantly (24). Despite calls for aggressive local therapy in these patients, the significance of treating isolated lymph node metastases remains controversial. Some have hypothesized that this is an intermediary stage prior to diffuse metastases and that treatment might delay or even prevent distant dissemination (28, 29). Others have hypothesized that lymph node metastases represent a distinct metastatic lineage, completely separate from bone or visceral metastases (30, 31). Those who believe that lymph node metastases are an intermediate step prior to widespread disease would argue for metastasis directed therapy while those who subscribe to the hypothesis that lymph nodes metastases are a distinct metastatic lineage believe there is likely little rationale or clinical benefit to treating lymph node metastases. In practice, distinct patterns of spread are likely not mutually exclusive and multiple patterns of metastatic seeding may occur (32). Additionally, both patterns of spread likely harbor castrate resistant clones which when treated may prolong a patient's response to ADT.

## Defining Oligo-Recurrent Nodal Disease

Despite a growing number of publications and even consensus statements there is no consistent definition for oligo-recurrent prostate cancer (33). Given that lymph node only prostate cancer may represent a favorable biology distinct from those with bone and visceral involvement, and that it certainly confers an improved prognosis, the authors of this paper argue against proposing a strict numerical cut-off for the number of metastases to qualify for oligo-recurrent nodal disease. Instead we are advocating for any volume of nodal disease that can be safely treated with curative intent. This approach preserves the rationale for treating nodal recurrent disease, which is to halt or delay widespread disease progression, while maximizing the number of patients who may benefit (28, 29). Because the potential benefit for treating nodal disease is diminished if cancer has already spread to the bone or visceral sites, one must be relatively certain no other systemic disease is present (34). For this reason, we recommend oligo-recurrence only be defined with the use of advanced PET imaging, preferably PSMA-PET, if available, which has been shown to outperform other agents in this clinical setting (24, 35).

## Overview of Local Therapy Treatment Options

The intent of ADT in this patient population is to delay progression and all patients with metastases not treated with localized therapy will ultimately progress. Successful treatment of nodal oligo-metastatic disease relies on treating all the involved nodes (34). Recently, data is emerging that localized therapy in the form of surgery or radiotherapy may be effective in treating these nodal oligo-recurrences. With evidence accumulating, 75% of panelists at the most recent Advanced Prostate Cancer Consensus Conference (APCCC) recommend systemic therapy and local treatment of all lesions for the majority of men with oligo-recurrent prostate cancer (33). While the optimal local therapy is an area of active clinical investigation, below we will review the most common types: surgery and radiation therapy.

## Salvage Lymph Node Dissection

Surgery in the form of salvage pelvic lymphadenectomies (sLND) may be beneficial for patients with hormone sensitive oligo-recurrent nodal disease (36). Studies have shown that sLND can delay clinical progression in up to 40% of patients with prior prostatectomy, with a subset potentially cured (37). Unfortunately, imaging is not yet sensitive enough to detect very small (< 4–5 mm) involved nodes, requiring sLND expansions beyond the PET positive fields (23, 38, 39). Despite these extensive interventions, many patients develop a second biochemical failure 1–2 years after sLND (37, 40). Additionally, sLND is technically challenging with a risk of significant complications requiring surgical management (41). Given the lack of durable response, technical difficulty and risk of morbidity from sLND, it continues to be recommended only on an experimental basis for a highly selective cohort of patients who are most likely to benefit from the procedure and high-level evidence is still missing to draw any clinically meaningful conclusion about the oncological impact of salvage lymph node dissection on long-term outcomes (36, 42).

## Radiation Therapy

**Figure 1:**
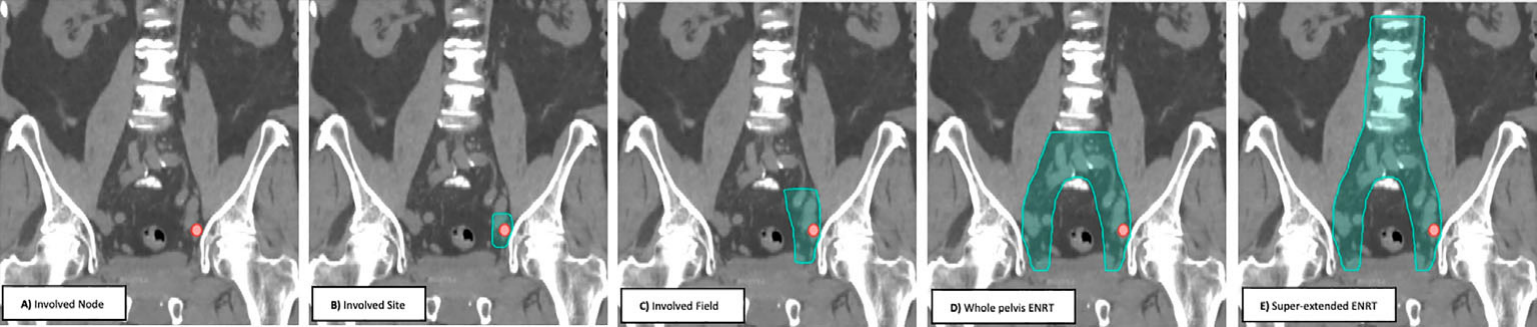
Nodal radiation therapy treatment volumes.

Prospective trials and retrospective multi-institutional reviews have used several radiotherapy treatment approaches (**Figure 1**) ranging from involved node (focal) stereotactic body radiotherapy (SBRT), involved site SBRT, involved field conventionally fractionated radiotherapy, whole pelvis elective nodal conventionally fractioned radiotherapy +/- a boost to gross disease (ENRT) and super-extended elective nodal radiotherapy (super-extended ENRT) (43). With the available data, no consensus treatment approach has emerged, with each approach having potential benefits and drawbacks (43). Below, we will review each of the treatment approaches with available data.

## Whole Pelvis Elective Nodal Radiation Therapy and Super-Extended Elective Nodal Radiation Therapy

Multi-institution reviews have suggested that involved node SBRT is safe and effective in treating nodal oligo-recurrences (44). However, several questions remain given the lack of long-term data compared to more conventional radiation therapy (45). Further, recurrences following more focalized SBRT generally occur in the adjacent, untreated pelvic or retroperitoneal lymph nodes (46). Because of this, some groups have advocated for elective nodal radiotherapy to consensus nodal volumes (ENRT) to reduce the risk of regional recurrences. This treatment strategy has been applied to patients with lymph node positive prostate cancer in the definitive and adjuvant setting and with long term follow-up has been found to be well tolerated (47). Because of the larger volumes being treated, ENRT is generally delivered in 25–30 small fractions (1.8–2 Gy/fx) (45). Retrospective data suggests that ENRT may decrease recurrences in patients with a solitary lymph node recurrence when compared to involved node SBRT, albeit at the cost of increased toxicity (45). A major limitation to this approach includes the increase of atypical patterns of lymphatic spread following primary treatment which consensus contouring atlases may omit in >50% of cases and which may necessitate an increase in field size (super extended-ENRT) to include the para-aortic, pre-sacral and peri-rectal regions, among others (48). Additionally, with a low (*α* / *β*) ratio, prostate cancer generally responds best to a relatively higher dose per fraction, which is limited with an ENRT or super-extended ENRT approach.

## Focal or Involved Node Stereotactic Body Radiation Therapy

For patients with limited metastatic sites, SBRT to the oligo-metastases may offer long-term local control and increased progression free survival (49). SBRT accurately delivers high doses to the target while sparing adjacent normal tissues (50). SBRT may improve tumor control and reduce treatment-related toxicity through improved target accuracy (51). This allows for high-dose (> 5 Gy per fraction), extremely hypofractionated treatment courses (1–5 fractions) that may be more radiobiologically effective and are certainly more convenient for patients than conventionally fractionated radiotherapy (51). Because of the high dose per fraction, SBRT targeting just the gross recurrent disease (focal or involved node SBRT) has been most extensively studied. Several recent clinical trials have confirmed the benefit of involved node (focal) SBRT to treat oligo-recurrent prostate cancer (34, 52, 53). The STOMP Trial randomized asymptomatic oligo-recurrent (≤ 3 sites) prostate cancer patients to observation or metastasis directed therapy (MDT) (metastasectomy or involved node (focal) SBRT) (52). The primary endpoint was ADT-free survival. ADT was initiated in both arms at time of symptoms, local progression or poly-metastatic disease (> 3 metastatic lesions). The median ADT-free survival was 13 months for the surveillance group and 21 months for the treated group. No grade 2 or higher toxicities were observed. More recently, the ORIOLE Trial was published which randomized asymptomatic oligo-metastatic (≤ 3 sites) prostate cancer patients to observation or involved node (focal) SBRT (19.5 Gy to 48 Gy in 3–5 fractions) (34). Progression at 6 months occurred in 61% of the patients in the observation arm but only 19% in the treated group. With increased follow-up of the STOMP trial, data is emerging that patients treated with MDT may experience increased time to castrate resistance (54). Criticisms of these trials include the lack of control arm of immediate ADT and the use of the surrogate end point ADT-free survival, however, for older men, with limited life expectancy this remains a clinically meaningful endpoint.

## Involved Site and Field Stereotactic Body Radiation Therapy

Most studies until this point have focused on involved node (focal) SBRT or ENRT, without significant investigation of alternative treatment volumes or synergy with systemic therapies. Additional treatment volumes include involved site and involved field radiotherapy (please refer to **Figure 1** for a graphical representation). These volumes expand on involved node to include an expansion on the gross disease to cover for adjacent microscopic disease. The rationale for these approaches stem from the subsequent regional nodal failures often seen when patients are treated with involved node SBRT.

Involved site radiotherapy includes a pre-specified expansion along the lymphatic nodal basin, while involved field generally involves an expansion to include the involved nodal basin as well as adjacent nodal basins. There is limited data on patterns of recurrence for involved site SBRT. Kneebone et al, noted distant lymph node recurrences as the most frequent site of recurrence without specific mention to their precise locations (55). To the authors' knowledge there has been no published data on involved field SBRT. Soldatov et el. used conventionally fractionated involved field radiotherapy in patients with oligo-recurrent nodal prostate cancer. The authors of this study noted relatively few adjacent or contralateral pelvic nodal failures using this treatment strategy but a relatively high rate of infield recurrences, perhaps owing to the small dose/fraction used in the study (56). Given the radiobiological advantages of SBRT, it is reasonable to believe that when combined with an involved field treatment volume, the rate of local control could improve.

## Proposed Treatment Volumes, Dose, and Fractionation Scheme For Involved Field Stereotactic Body Radiation Therapy

**Figure 2:**
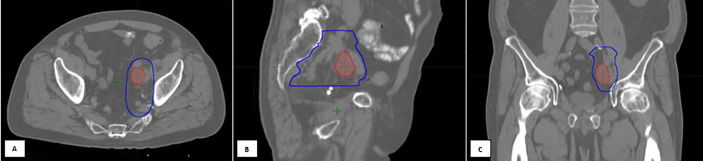
An 80-year-old gentleman with oligo-recurrent prostate cancer with left external iliac adenopathy identified on PET imaging [**(A)** Axial, **(B)** Sagittal, **(C)** Coronal views]. The decision was to proceed with SBRT assisted Intermittent ADT. An involved field SBRT approach was utilized. The left external iliac, left obturator, and left internal iliac nodal regions were treated with 27.5 Gy in 5 fractions (high risk CTV), with a 30 Gy simultaneous integrated boost to the gross disease. Treatment planning computed tomography images showing the isodose lines for the GTV prescription of 30 Gy (red) and the High-risk CTV prescription of 27.5 Gy (dark blue line). Following SBRT, induction ADT was initiated.

**Figure 3:**
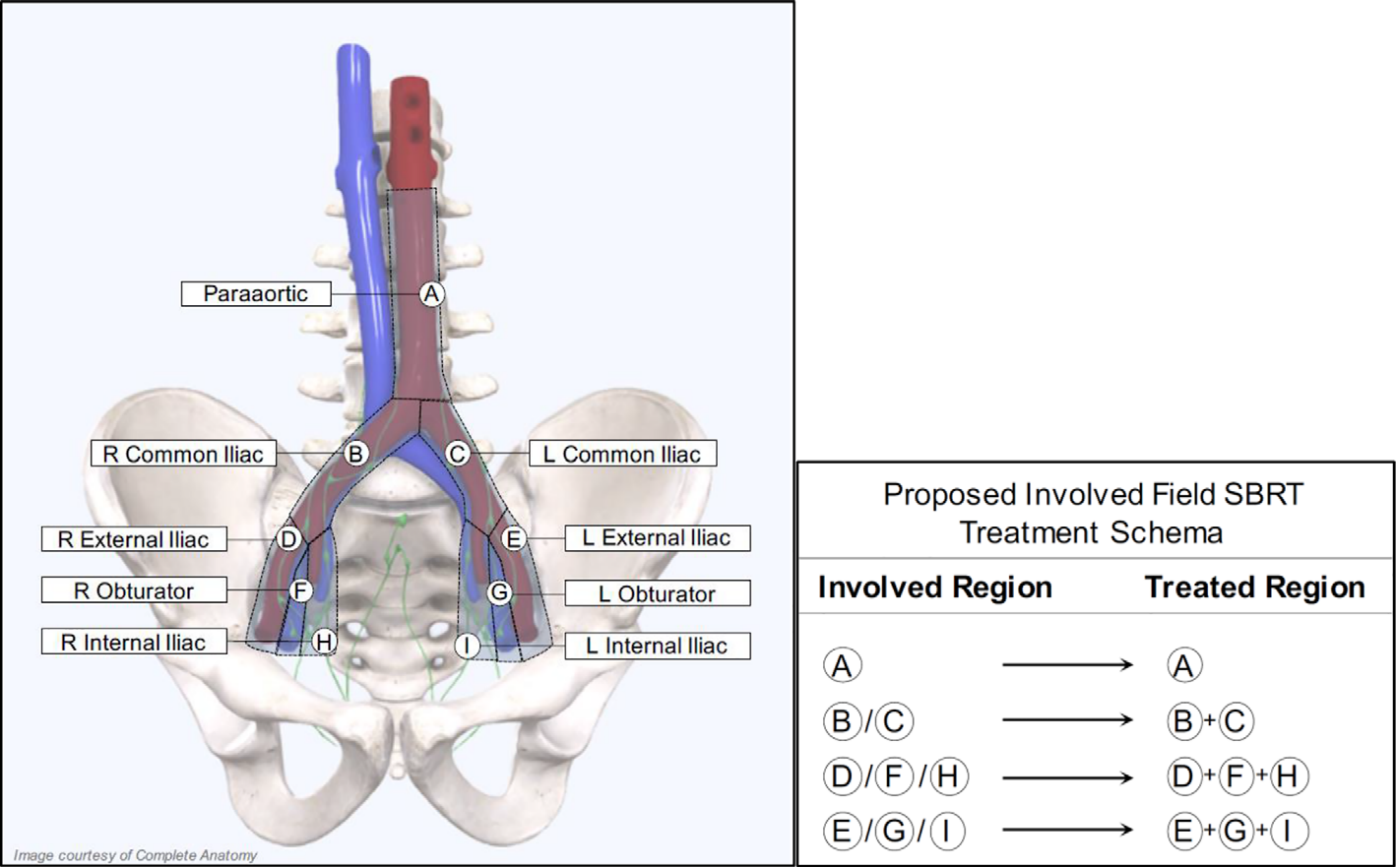
Proposed treatment volumes for Involved Field SBRT.

For patients receiving salvage radiation for oligo-recurrent nodal prostate cancer, the optimal systemic therapy, radiation dose, fractionation schedule, and treatment volume remain unanswered questions. Given the potential radiobiological advantages, improved local control, increased patient convenience and the poor coverage of consensus treatment atlases utilizing ENRT the authors of this paper advocate for treatment of nodal metastases with a form of involved field SBRT. This treatment approach has several distinct advantages over the other commonly utilized treatment options and at the very least, requires further exploration. First, it expands on the gross disease to include the adjacent nodes where an involved node SBRT approach is most likely to fail. Further, because this approach utilizes relatively small treatment volumes, it allows for safe dose escalation and hypo-fractionation, improving local control and patient convenience. Additionally, from the available literature, treatment with involved field has a low rate of contralateral pelvic nodal failure, providing a rationale for the omission of these volumes in many situations (56). Further, because of the conformality and dose fall of from SBRT, if a patient were to fail in the contralateral pelvis, these could be salvaged with additional involved field SBRT, without a significantly increased risk of toxicity. Lastly, and perhaps most importantly, many of these patients will eventually progress regardless of the type of local therapy used, so it is critically important to minimize treatment burden in this population. **Figure 2** provides an example of involved field utilized for the treatment of a patient with oligo-recurrent prostate cancer to the left external iliac lymph node. **Figure 3** shows our proposed treatment volumes for pelvic nodal oligo-recurrences.

No optimal SBRT dose regimen has been established due to the variation in target volume and proximity to normal structures (57). Retrospective data suggests that a biologically effective dose (BED) ≥100Gy (assuming an *α* / *β* = 3) is needed for adequate long-term local control following SBRT (39). Because of the need to balance safety with efficacy, we hypothesize that treating the gross nodal disease to 30 Gy/5 fx with a simultaneous high-risk CTV expansion of 27.5 Gy/5 fx to the adjacent nodal basin(s) allows for high rates of local control with minimal toxicity. Because the BED of this radiation scheme is less than 100 Gy (BED = 88 Gy, (*α* / *β* = 3), the threshold that retrospective data suggests is needed for favorable long-term local control, it needs to be given concurrently with a radiosensitizer. Here, we plan to utilize ADT, which has been shown to increase the effectiveness of radiation by decreasing prostate cancer specific DNA repair mechanisms, allowing for the use of lower radiation doses without sacrificing local control (58). We hypothesize that this treatment dose and fractionation, when combined with concurrent ADT, will allow for high rates of long term local control with minimal acute and late toxicity. This strategy also allows for the safe and effective treatment of para-aortic nodal metastases, which has been limited due to the sensitivity of the adjacent small bowel to radiation (59). Our early experience with our proposed approach suggests that prostate SBRT will not adversely affect this (49, 60).

## Proposed Dose Constraints

We hypothesize that involved field SBRT will decrease tumor burden in lymph nodes and delay transition to bone metastases and castration resistance (54). However, if involved field SBRT to oligo-recurrent nodal disease causes a significant rate of high-grade late toxicity and/or adversely affected patients' long-term quality of life this approach would not be worth pursuing further. Data on the dose tolerance of the bowel to SBRT is limited (61). It is clear that high doses of radiation to small portions of the bowel can cause significant complications (≥ grade 3) (62). Currently, the impact of SBRT to oligometastatic disease on overall survival is unknown. Therefore, we recommend a very conservative constraint of 30 Gy <1 cc to the bowel, a constraint for which the risk of significant toxicity is very low (5%) (63). Moderate doses to larger portions of bowel may also impact the risk of toxicity so we recommend the volume of bowel receiving 20 Gy <15 cc (64). (Please see **Table 1** for additional proposed dose constraints).

**Table 1:** Proposed Dose Constraints for OARs when treating gross nodal disease to 30 Gy/5 fx with a simultaneous high-risk CTV expansion of 27.5 Gy/5 fx.

Involved Field SBRT Dose Constraints for Organs at Risk (OARs)	
Organ at Risk	Constraints
Bowel	V20 Gy < 15 cc
	V30 Gy < 1 cc
	V35 Gy < .1 cc
Kidney	At least 200 cc receiving less than 17.5 Gy
Liver	At least 700 cc receiving less than 21 Gy
Spinal Cord	Dmax < 30 Gy
	V23 Gy < .35 cc
Stomach	Dmax < 30 Gy
	V18 Gy < 10 cc
Rectum	Dmax < 30 Gy
	V25 Gy < 10 cc
Ureter	Dmax < 40 Gy
Bladder	Dmax < 30 Gy
	V18 Gy < 10 cc
**Table 1**	

## A Cycle-Based Treatment Approach

### PSA response to ADT as a Prognostic Tool

Early PSA response to ADT and other therapies is prognostic (9, 21, 65). In general, optimal PSA suppression takes 6–9 months (66). A PSA ≤0.2 ng/ml after 7 months of ADT suggests a long median survival (21, 65). Here, we plan to utilize induction ADT as a prognostic tool to determine if a patient would benefit from more intensive systemic therapy or if they would be reasonable candidates for an intermittent treatment approach such as this. We therefore recommend a conservative, risk adaptive approach for determining the length of induction ADT and which patients would be candidates for continued I-ADT. Following involved field SBRT, we recommend a 3-month induction period where all patients receive ADT. At 3 months, we recommend checking PSA and testosterone levels, if the PSA is ≤0.2 ng/ml, the PSA level that is associated with improved prognosis, then hold further ADT and continue to follow PSA and testosterone levels every 3 months. However, if at 3 months the PSA fails to fall ≤0.2 ng/ml, we recommend an additional 3-month induction period of ADT. If after an additional 3 months (6 months following the initiation of ADT) the PSA still fails to fall ≤0.2 ng/ml, this is indicative of a less favorable prognosis and we do not recommend the patient proceed further with this intermittent treatment strategy. Instead, these patients would likely benefit from continuous ADT with the possible addition of other systemic agents.

### Timing of Repeat Imaging and Re-Initiation of Androgen Deprivation Therapy

**Figure 4:**
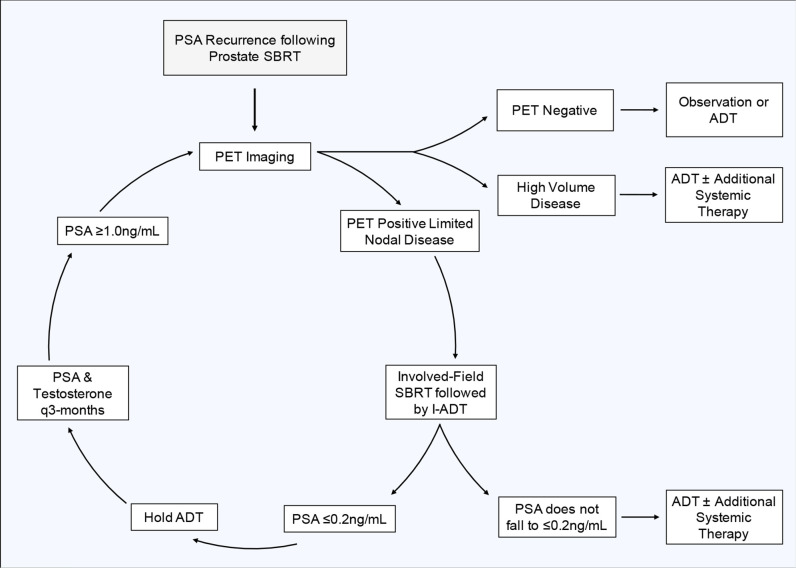
Involved Field SBRT assisted I-ADT Treatment Cycle.

Although a subset of patients may have a prolonged clinical response and may never need subsequent treatment, most patients will eventually relapse. From published patterns of recurrence following SBRT, the majority of patients re-present with ≤3 new metastases, allowing for subsequent MDT (46). For this reason, and because intermittent ADT requires frequent total testosterone and PSA checks, we recommend following these values every 3 months. If a patient's PSA starts to rise following a cycle of ADT and SBRT, we recommend repeat PET imaging when the patient's PSA rises to ≥1 ng/ml. At this PSA level, PSMA-PET has been shown to perform well in the detection (> 80%) of recurrent prostate cancer (67). While the performance of PET imaging improves with increasing PSA, this PSA cut off balances a relatively high rate of disease detection without allowing undue progression of disease that may sacrifice clinical outcomes (68). If imaging reveals new oligo-recurrent disease, we would treat the disease with another cycle of involved field SBRT and re-initiate ADT using the parameters previously outlined. **Figure 4** shows our proposed treatment cycle. If imaging reveals high burden metastatic disease, we recommend re-initiating ADT with the possible addition of other systemic agents.

## Conclusion

Recurrent prostate cancer remains a complex disease. ADT is successful in delaying the progression and improving overall survival. Unfortunately, castrate-resistant clones may be present early in the disease process even prior to the initiation of ADT, creating the need for alternative treatments. SBRT has been demonstrated as a safe and efficacious modality. Implementation of involved field SBRT early in the disease process may reduce overall tumor burden, in turn delaying progression of disease, prolonging response to intermittent ADT and improving both the quality and length of life. Strengths of this approach include: minimizing a patient's exposure to ADT and the side effects related to its receipt, utilizing large fraction sizes to capitalize on the low intrinsic (*α* / *β*) of prostate cancer to improve local control and short treatment courses that improve patient convenience and reduce indirect patient costs. Potential limitations of this approach include: adverse patient outcomes in those that may have benefited from more aggressive upfront systemic therapy and a potential sacrifice in long term local control with the delivery of less than a truly ablative dose of radiation in order to prioritize long term patient safety. In conclusion, the current cyclic approach balances the benefits of local treatment and limited ADT versus the potential benefit of long-term ADT in those with unfavorable features. The authors plan to test this treatment strategy in a single institution phase II clinical trial.

## Author Contributions

MC is the lead author who participated in manuscript drafting, table/figure creation, and manuscript revision. MLC, AP, EW, MF, and MM aided in the table/figure creation. LB and SL are the dosimetrists who contributed dosimetric data and figures. PL, RH, SC, DK, NA, GE, SS, BC, JL, and ND are senior authors who aided in drafting the manuscript and manuscript revision. SC is the corresponding author who initially developed the concept, and drafted and revised the manuscript. All authors contributed to the article and approved the submitted version.

## Funding

This work was supported by NIH Grant P30CA051008. We gratefully acknowledge the Grant No R01MD012767 from the National Institute on Minority Health and Health Disparities (NIMHD), NIH to DK and SC.

## Conflict of Interest

SP Collins and BT Collins serve as clinical consultants to Accuray Inc.

The remaining authors declare that the research was conducted in the absence of any commercial or financial relationships that could be construed as a potential conflict of interest.

## Abbreviations

ADT, androgen deprivation therapy; CRPC, Castrate-resistant prostate cancer; DVH, dose-volume histogram; GTV, gross target volume; IGRT, image-guided radiation treatment; LHRH, luteinizing hormone-releasing hormone; PSA, prostate-specific antigen; PTV, planning target volume; SBRT, stereotactic body radiation therapy; TURP, transurethral resection of the prostate.
